# Non‐lethal proteasome inhibition activates pro‐tumorigenic pathways in multiple myeloma cells

**DOI:** 10.1111/jcmm.14653

**Published:** 2019-09-30

**Authors:** Aikaterini Skorda, Aimilia D. Sklirou, Theodore Sakellaropoulos, Despoina D. Gianniou, Efstathios Kastritis, Evangelos Terpos, Ourania E. Tsitsilonis, Bogdan I. Florea, Herman S. Overkleeft, Meletios A. Dimopoulos, Leonidas G. Alexopoulos, Ioannis P. Trougakos

**Affiliations:** ^1^ Department of Cell Biology and Biophysics Faculty of Biology National and Kapodistrian University of Athens Athens Greece; ^2^ School of Mechanical Engineering National Technical University of Athens Athens Greece; ^3^ Department of Clinical Therapeutics School of Medicine National and Kapodistrian University of Athens Athens Greece; ^4^ Department of Animal and Human Physiology Faculty of Biology National and Kapodistrian University of Athens Athens Greece; ^5^ Gorlaeus Laboratories Leiden Institute of Chemistry and Netherlands Proteomics Centre Leiden The Netherlands

**Keywords:** cytokines, kinases, multiple myeloma, proteasome, proteasome inhibitors

## Abstract

Multiple myeloma (MM) is a haematological malignancy being characterized by clonal plasma cell proliferation in the bone marrow. Targeting the proteasome with specific inhibitors (PIs) has been proven a promising therapeutic strategy and PIs have been approved for the treatment of MM and mantle‐cell lymphoma; yet, while outcome has improved, most patients inevitably relapse. As relapse refers to MM cells that survive therapy, we sought to identify the molecular responses induced in MM cells after non‐lethal proteasome inhibition. By using bortezomib (BTZ), epoxomicin (EPOX; a carfilzomib‐like PI) and three PIs, namely Rub999, PR671A and Rub1024 that target each of the three proteasome peptidases, we found that only BTZ and EPOX are toxic in MM cells at low concentrations. Phosphoproteomic profiling after treatment of MM cells with non‐lethal (IC_10_) doses of the PIs revealed inhibitor‐ and cell type‐specific readouts, being marked by the activation of tumorigenic STAT3 and STAT6. Consistently, cytokine/chemokine profiling revealed the increased secretion of immunosuppressive pro‐tumorigenic cytokines (IL6 and IL8), along with the inhibition of potent T cell chemoattractant chemokines (CXCL10). These findings indicate that MM cells that survive treatment with therapeutic PIs shape a pro‐tumorigenic immunosuppressive cellular and secretory bone marrow microenvironment that enables malignancy to relapse.

## 1 INTRODUCTION

The integrity of proteome homeodynamics (proteostasis) is critical for cell homeostasis and survival and is maintained by the concerted action of several modules that constitute the proteostasis network (PN). Proteostasis network is a multi‐compartmental highly wired system, which co‐ordinates protein synthesis, folding, trafficking, disaggregation and degradation.^1^, ^2^, ^3^ A key component of the PN and a module for degradation of polypeptides is the ubiquitin‐proteasome pathway (UPP). Ubiquitin‐proteasome pathway is composed from the ubiquitin‐conjugating enzymes and the 26S proteasome; it is the site of protein synthesis quality control and is involved in the degradation of both normal short‐lived polypeptides and of misfolded or unfolded proteins.^4^ Polypeptide hydrolysis is catalysed by three peptidase sites located in the β1, β2 and β5 20S proteasome subunits, which bear caspase (C‐L)‐, trypsin (T‐L)‐ and chymotrypsin (CT‐L)‐like activities, respectively.^4^, ^5^

The imperative necessity of polypeptides to obtain their proper three‐dimensional structure lies on the fact that they essentially are parts of complex protein machines, which are involved in virtually every cellular function, including genome stability and repair. In support, PN malfunction has been associated with numerous diseases, including cancer.^1^, ^6^ As over‐activation of the proteostatic modules represents a hallmark of advanced tumours,^1^, ^7^ their inhibition provides a strategy for the development of novel antitumour therapies. Consistently, therapeutic targeting of the proteasome peptidases activities is currently approved for the treatment of multiple myeloma (MM) and mantle‐cell lymphoma (MCL) and remains a challenge for the cure of solid tumours.^8^, ^9^

Ubiquitin‐proteasome pathway inhibitors, which have demonstrated clinical antitumour therapeutic efficacy, include bortezomib (BTZ), carfilzomib (CFZ) and ixazomib.^8^, ^9^ BTZ, the first proteasome inhibitor (PI) approved for clinical use, is a slowly reversible inhibitor that binds the catalytic site of the 26S proteasome enabling inhibition of the CT‐L and to a lesser extent of C‐L and T‐L activities.^10^, ^11^ CFZ is a second‐generation irreversible PI that specifically targets the CT‐L activity and is administrated in patients with relapsed or refractory MM^9^; epoxomicin (EPOX) is a CFZ‐like irreversible PI which served as a scaffold for CFZ generation.^12^

MM is a plasma cell neoplasm that accounts for ~2% of all haematological malignancies and is characterized by clonal plasma cell proliferation in the bone marrow.^13^ Although recent developments in the treatment of MM have led to significant improvements in response rates and overall survival, resistance to PIs and relapse are inevitable in almost all patients and remain a burden in MM therapy.^14^, ^15^ This outcome, apart from referring to MM cell clones with innate or acquired drug resistance, may also relate to MM cells that survive the therapeutic cycles with PIs due to minimal proteasome inhibition that was not sufficient to promote apoptosis. Therefore, the necessity for improved therapeutic treatments along with the *in‐depth* understanding of the triggered molecular responses in the tumour with a focus on those cells that survive therapy with PIs is urgent.

To address this issue, we studied the short‐ and long‐term effects induced by non‐lethal (IC_10_) doses of distinct classes of PIs, namely BTZ, EPOX and of three highly selective PIs (Rub999, PR671A and Rub1024) in the ΜΜ cell lines JJN3 and RPMI 8226. We performed phenotypic analyses along with phosphoproteomic and cytokine/chemokine profiling by using the xMAP technology. Our findings revealed that non‐lethal doses of PIs activate pro‐survival pathways in MM cells leading to secretion of pro‐tumorigenic immunosuppressive cytokines/chemokines that likely enable disease progression.

## 2 MATERIALS AND METHODS

### 2.1 Cell lines and cell culture conditions

The human MM cell lines JJN3 and RPMI 8226 were kindly provided by Prof. C. Mitsiades (Dana‐Farber Cancer Institute, Harvard Medical School, Boston, USA) and maintained in RPMI 1640 medium (Biosera) containing 10% foetal bovine serum (Thermo Fisher Scientific), at 5% CO_2_, 37°C.

### 2.2 Proteasome inhibitors

BTZ (PS‐341) was from Calbiochem and EPOX from Enzo Life Sciences. BTZ and EPOX were diluted in distilled water and DMSO, respectively, and were stored at −20°C. Rub1024 (NC‐001),^16^ PR671A (LU102)^17^ and Rub999 (NC‐005)^16^ were produced by chemical synthesis; reportedly, their inhibitory effect is exerted at the C‐L, T‐L and CT‐L proteasomal activities, respectively. Rub1024, PR671A and Rub999 were diluted in DMSO and stored at −20°C.

### 2.3 MAPK, STAT and MTH1 inhibitors

The MAPK inhibitors CI‐1040 (against MEK 1/2) and JNK‐IN‐8 (against JNK 1/2/3) were obtained from Cayman Chemical and Sigma‐Aldrich, respectively. The MTH1 inhibitor TH588 was a kind offer from Prof. T. Helleday (Karolinska Institutet, Solna, Sweden). The STAT inhibitors Stattic (against STAT3) and AS1517499 (against STAT6) were purchased from Sigma‐Aldrich. Inhibitors were diluted in DMSO and stored at −20°C.

### 2.4 Cell viability and measurement of proteasome peptidase activities

The cytotoxic effect of PIs against the MM cell lines was determined by using the MTT reagent (Sigma‐Aldrich). The proteasome activities were measured as described before.^18^ For details, see also Supporting Information.

### 2.5 Cell treatment with PIs and measurement of phosphorylated proteins and secreted cytokines/chemokines using xMAP technology

Cells were plated in flat‐bottomed 12‐well plates at a concentration of 500 000 cells/mL in the presence (or not) of PIs, and plates were transferred in a humidified incubator (37°C); 24‐48 hours later, the samples corresponding to day 1 and day 2 of treatment were collected. At day 3 (72 hours), cells were counted and plated in flat‐bottomed 12‐well plates at a concentration of 500 000 cells/mL, in the presence of fresh medium containing the selected concentration of PIs. At day 6 (144 hours), cells were treated as in day 3. Finally, at day 7 (168 hours) samples were collected for downstream analyses.

Collected cell cultures' material (cells and culture medium) was centrifuged at 3000 *g* for 5 minutes. Supernatants containing the secreted cytokines/chemokines were kept at −80°C. For the isolation of phosphoproteins, cells were washed with 200 μL of phosphate‐buffered saline (PBS) and were lysed using 60 μL of suitable lysis buffer supplemented with protease and phosphatase inhibitors. Lysates were centrifuged at 13 300 *g* (4°C), and the supernatants were used to determine protein concentration by Bradford assay; samples were stored at −80°C until the acquisition of all time‐points. For the implementation of the bead‐based sandwich enzyme‐linked immunosorbent assay (ELISA) protocol, 50 μL of xMAP magnetic beads coupled with specific antibodies (1700 beads per well/each protein) was placed in flat‐bottomed 96‐well plates. Then, 50 μL of a cell lysate or supernatant was incubated with the beads for 1.5 hours in order to capture the target proteins. Following two wash steps with 100 μL of 1% BSA‐PBS solution, beads were incubated for 1 hour with 20 μL of detection antibodies coupled with biotin. Subsequently, 50 μL of streptavidin‐phycoerythrin (PE) solution (5 μg/μL) was added, and after 15 minutes of incubation, the beads were washed and re‐suspended in 130 μL of 1% BSA‐PBS solution. Measurements were performed using a FLEXMAP 3D Luminex system, and results were processed with MATLAB software.

### 2.6 Statistical analysis

Experiments were performed at least in duplicates, and shown data points correspond to the mean of independent experiments. Statistical analysis was performed by using the MS Excel and the Statistical Package for Social Sciences (IBM SPSS; version 19.0 for Windows); significance was evaluated using one‐way analysis of variance (ANOVA). Error bars indicate standard deviation (SD); significance at *P* < .05 or *P* < .01 is indicated in graphs or heatmaps by one or two asterisks, respectively. Significance for the phosphoproteomic and cytokine/chemokine secretion set of experiments was estimated as a combination of median fluorescence intensity (MFI) value above 600 and fold change (FC) value above 0.3 when compared to control samples.

Additional methods are available in Supporting Information.

## 3 RESULTS

### 3.1 BTZ and EPOX induce cell death and suppress proteasome peptidases activity in MM cell lines at relatively low concentrations

First, we examined the effect of BTZ and EPOX on the survival of JJN3 and RPMI 8226 cell lines. As shown in Figure S1A, both BTZ and EPOX induced extensive cell death in JJN3 cells even at very low concentrations (BTZ IC_50_, 3.99 nM; EPOX IC_50_, 7.40 nM). The RPMI 8226 cells were relatively more resistant (as compared to JJN3 cells) to BTZ (IC_50_, 5.5 nM) and especially to EPOX (IC_50_, 18 nM; Figure S1B). Under these experimental conditions, the IC_10_ values for the JJN3 cell line were 2.45 nM for BTZ and 4.54 nM for EPOX, while for RPMI 8226 were 1.8 nM for BTZ and 5.5 nM for EPOX.

Furthermore, we investigated the extent by which cell exposure (for 24 or 48 hours) to BTZ or EPOX at either IC_10_ or IC_50_ concentrations affects proteasome activities. As shown in Figure S1C, BTZ inhibited mostly CT‐L and C‐L peptidases in JJN3 cells at both IC_10_ and IC_50_ doses, while EPOX was more selective for the CT‐L activity, although it also affected C‐L and T‐L activities (Figure S1C). Similarly, BTZ was found to inhibit mainly CT‐L and C‐L activities in RPMI 8226 cells, whereas EPOX was more selective for CT‐L activity (Figure S1D). Inhibition of proteasome activities upon incubation with PIs was more intense in JJN3 than in RPMI 8226 cells. Collectively, BTZ and EPOX inhibited C‐L and T‐L activities in addition to CT‐L activity and showed high toxicity against JJN3 and RPMI 8226 cell lines.

### 3.2 Among PIs that are highly selective for specific proteasomal peptidases, only Rub999 was partially toxic against MM cells

We then assayed the effects of Rub999, Rub1024 and PR671A inhibitors on MM cells. We found that Rub1024 or PR671A was (after treatment for 24 hours) not toxic in JJN3 or RPMI 8226 cells (Figure S2A,B), while Rub999 induced significant cell death in both MM cell lines but at higher concentrations as compared to BTZ or EPOX (IC_50_ = 150 nM for JJN3 and 180 nM for RPMI 8226; Figure S2A_1_,B_1_); a comparative summary of cell viability after exposing JJN3 and RPMI 8226 cells to different doses of the studied PIs is shown in Figure S3.

Rub999, Rub1024 and PR671A specificity against proteasome peptidases was tested at the concentrations of 50 nM of Rub999 and 500 nM of Rub1024, while PR671A was used at 500 nM for JJN3 and 800 nM for the RPMI 8226 cells. As reported before,^16^, ^17^ we noted that for both JJN3 (Figure S2C) and RPMI 8226 (Figure S2D) cells, the Rub999, Rub1024 and PR671A inhibitors selectively suppressed the CT‐L, C‐L and T‐L activities, respectively. Thus, as was suggested,^19^, ^20^, ^21^ the high percentage of cell death in MM cells achieved by BTZ and EPOX (and likely CFZ) is associated with co‐inhibition of more than one proteasomal peptidases. These findings also support the notion that the CT‐L activity is the rate limiting for protein breakdown and accordingly, selective inhibition of the β5 peptidase by Rub999 increased cell death, yet, as mentioned less effectively and at higher concentrations as compared to BTZ or EPOX, and likely CFZ.^22^

### 3.3 Proteasome inhibition at non‐lethal doses in MM cells modifies cell signalling in an inhibitor‐, time‐ and cell type‐specific manner; it also induces pro‐tumorigenic and/or immunosuppressive signalling pathways

It was suggested that short incubation times of 1‐2 hours with high PI concentrations (eg 250 nM) can be used in cell‐based assays to mimic the therapeutic intervention in the clinic.^23^ Yet, herein we aimed to avoid extensive tumour cell death in order to investigate the effects of the PIs after partial non‐lethal proteasome inhibition that could likely contribute to tumour relapse. To this end, we treated MM cells with IC_10_ doses of BTZ and EPOX; under these conditions, proteasome peptidases are inhibited without extensive cell death (Figure S1). The Rub999, Rub1024 and PR671A PIs were added at the concentrations determined by the proteasome activity assays shown in Figure S2C,D. Thus, the measured output mostly relates to proteasome moderate inhibition‐mediated cell responses (eg phosphorylation and/or secretion of key molecules) and not to apoptotic events. Cells were treated with fresh medium containing the PI every 72 hours, and samples were collected at days 1, 2 and 7 for the BTZ and EPOX analyses; or at days 1 and 2 for Rub999, Rub1024 and PR671A.

**Figure 1:**
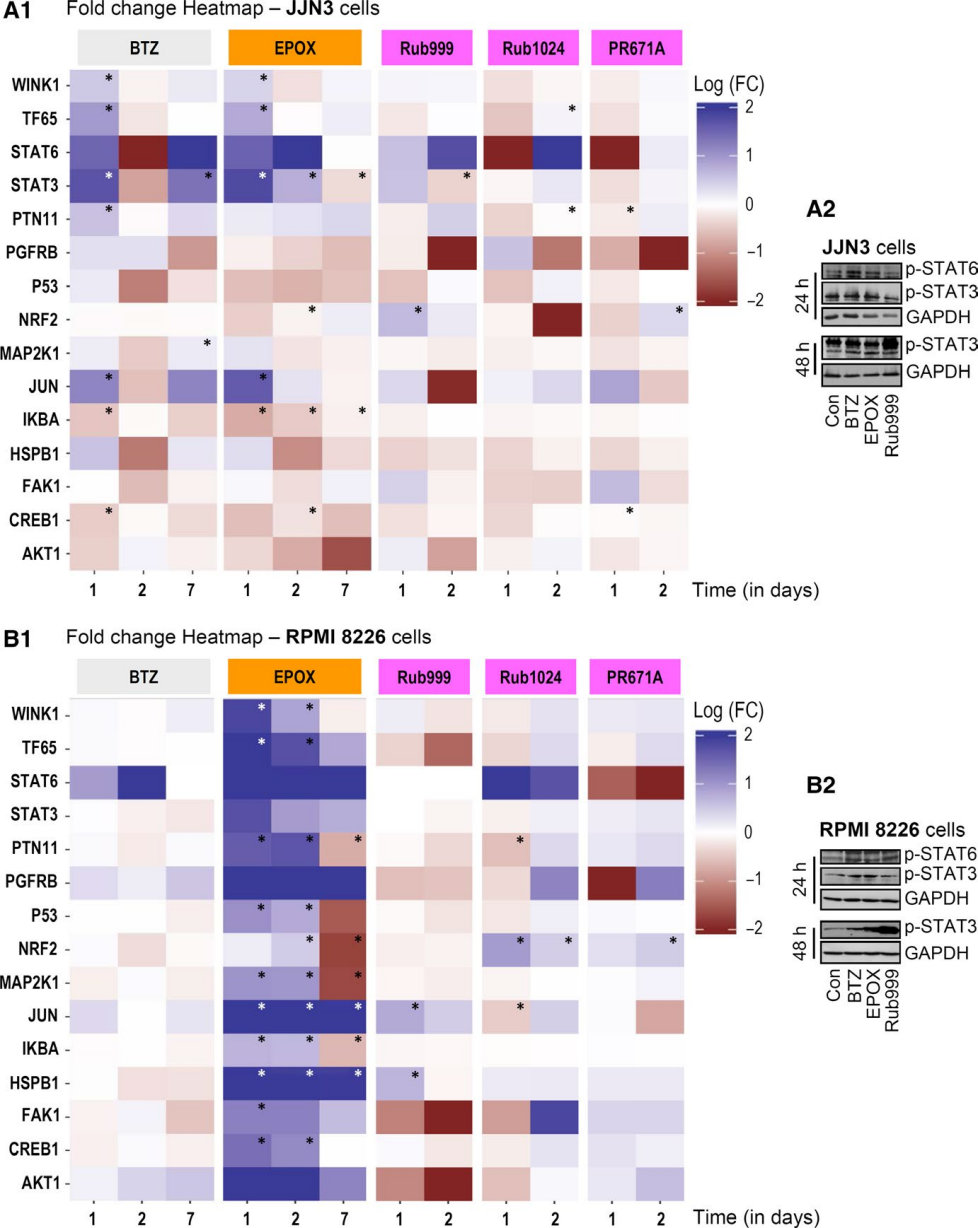
Non‐lethal inhibition of proteasome in MM cells activates pro‐survival, tumorigenic and immunosuppressive pathways in an inhibitor‐ and cell type‐specific manner. A_1_, Heatmap indicating logarithmic fold change (FC) values (vs control samples) of the basal phosphorylation levels of the shown proteins after incubating JJN3 cells with either BTZ and EPOX for 24, 48 and 168 h or with Rub999, Rub1024 and PR671A for 24 and 48 h. A_2_, Representative immunoblotting analyses of JJN3 cell protein samples probed with antibodies against p‐STAT3 and p‐STAT6 after exposure of cells to the shown PIs for 24 or 48 h. B_1_, Heatmap indicating logarithmic FC values (vs control samples) of the basal phosphorylation levels of the shown proteins in RPMI 8226 cells incubated with either BTZ and EPOX for 24, 48 and 168 h or with Rub999, Rub1024 and PR671A for 24 and 48 h. B_2_, Representative blots showing STAT3 and STAT6 phosphorylation levels after exposure of RPM1 8226 cells to the shown PIs for 24 or 48 h. Significance (*) of the results in (A_1_, B_1_) was set as a combination of median fluorescence intensity (MFI) value above 600 and FC value above 0.3 vs control samples. Probing with GAPDH in (A_2_, B_2_) was used as total protein loading reference

For these analyses, we used a bead‐based sandwich ELISA protocol followed by plate measurement using the LUMINEX platform for a phospho‐panel of 15 proteins (Table S1). We found that exposure of MM cells to PIs resulted in PI‐, time‐ and cell type‐specific readouts (Figure 1); data were also tested for their significance (asterisks in Figure 1A_1_,B_1_), plotted on bar graphs (Figure S4) and used for hierarchical clustering (HCL) analyses (Figure S5). Treatment of JJN3 cells with BTZ tended to increase at day 1 the phosphorylation of most of the assayed proteins (except IKBA, CREB1 and AKT1); these changes were mostly inverted or ceased at day 2, while the phosphoprotein profile at day 7 was in most cases similar to day 1. Similarly, 24‐hour (day 1) exposure of MM cells to EPOX resulted in increased phosphorylation levels of targeted proteins and it suppressed the phosphorylation of P53, NRF2, IKBA, CREB1 and AKT1. This pattern was mostly retained at day 2 (except for WNK1, MAP2K1 and HSPB1), whereas these responses were significantly milder at day 7 except for a strong suppression of AKT1 phosphorylation. Rub999 showed a phosphorylation profile more similar (yet milder) to BTZ, while the noted alterations for Rub1024 and PR671A were weaker except for STAT6 and PGFRB reduced phosphorylation at day 1 and NRF2 suppressed phosphorylation (Rub1024) at day 2 (Figure 1A_1_; Figures S4A and S5A). At the relatively more resistant to the PI RPMI 8226 cells, BTZ exerted minor alterations (except for STAT6 increased phosphorylation at day 2), while EPOX caused increased phosphorylation of most assayed proteins, except for P53, NRF2, MAP2K1 and IKBA at day 7. Rub999 strongly suppressed the phosphorylation levels of TF65, FAK1 and AKT1; Rub1024 increased STAT6 phosphorylation (which was suppressed by PR671A) and both Rub1024 (day 1) and PR671A (day 2) increased NRF2 phosphorylation (Figure 1B_1_; Figures S4B and S5B). Notably, in this cell line both the Rub1024 and PR671A PIs tended to induce the phosphorylation of the assayed proteins at day 2. Our findings for phospho‐STAT3 and phospho‐STAT6 were largely verified by immunoblotting analyses in JJN3 and RPMI 8226 cell lysates following treatment with the studied PIs (Figure 1A_2_,B_2_).

### 3.4 Combined treatment of MM cells with PIs and a MTH1 inhibitor induced synergistic effects, whereas co‐treatment of MM cells with PIs and STAT3, STAT6 or MAPK inhibitors only mildly increased cell death

**Figure 2:**
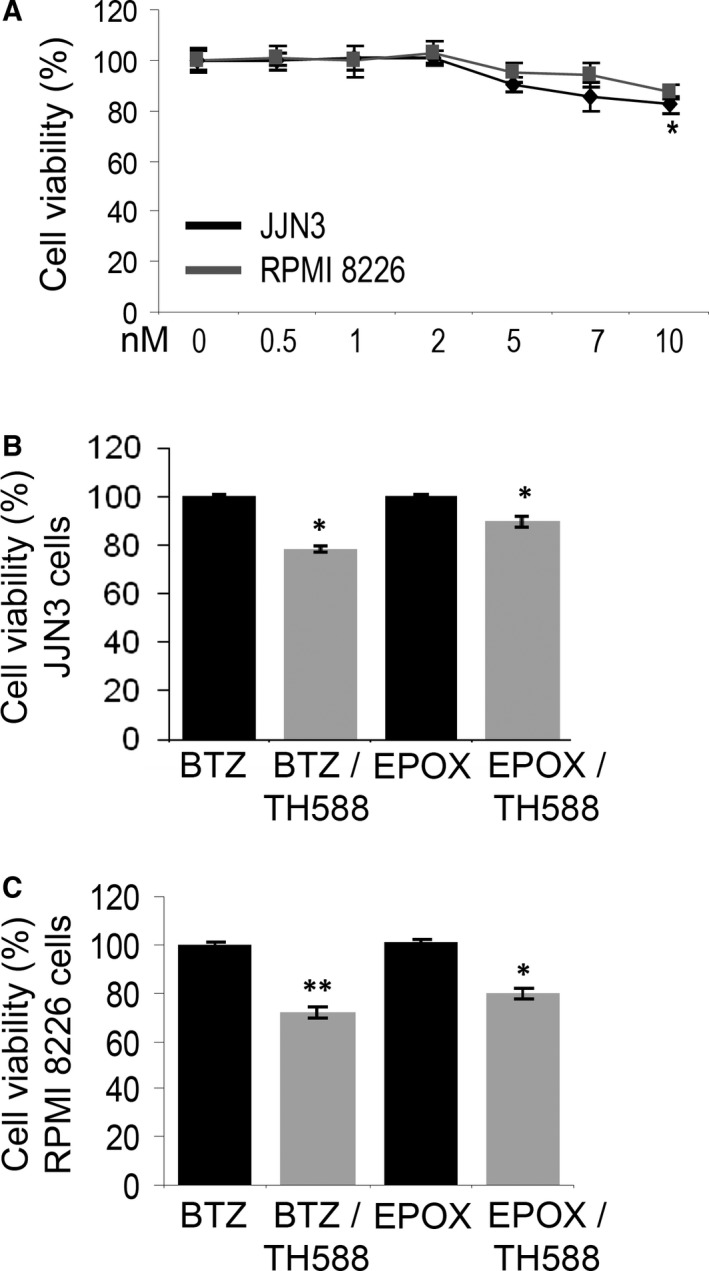
Combined proteasome and MTH1 inhibition exerted mild synergistic pro‐death effects on MM cells. A, Relative (%) viability of JJN3 and RPM1 8226 cell lines incubated with the MTH1 inhibitor TH588 for 24 h. B, C, Relative (%) survival of JJN3 (B) and RPM1 8226 (C) cell lines after a combinatorial treatment with BTZ or EPOX (at IC_10_ concentration) in the presence (or not) of the TH588 inhibitor for 24 h. BTZ (JJN3 cells, 2.45 nM; RPMI 8226, 1.8 nM), EPOX (JJN3 cells, 4.54 nM; RPMI 8226, 5.5 nM), TH588 (JJN3 cells, 5.5 μM; RPMI 8226, 9 μM). Bars: ± SD, *P < .05, **P < .01 vs controls set to 100%

As PIs induce oxidative stress,^24^, ^25^ we combined PI treatment with the MTH1‐specific inhibitor, TH588; the MTH1 enzyme hydrolyses oxidized nucleotides preventing thus their incorporation into the DNA.^26^ Exposure of MM cells to TH588 showed that these cell lines are relatively resistant to TH588 (Figure 2A). Yet, combined treatment for 24 hours of MM cells with TH588 and BTZ or EPOX at their IC_10_ concentrations caused a significant reduction in cell viability in both cell lines and for both PIs (Figure 2B,C).

Next, we examined whether co‐treatment of MM cells with low PI doses (IC_10_) and STAT3 (Stattic, a STAT3 INH) or STAT6 (AS1517499, a STAT6 INH) inhibitors enhance PI toxicity. We noted that both JJN3 and RPMI 8226 cells are more resistant (compared to STAT3) to STAT6 inhibition (Figures S6A and S7A). Combined treatment of JJN3 cells with EPOX and the STAT3 INH or with Rub999 and the STAT3 or STAT6 INHs for 24 hours only mildly increased PIs' cell death (Figures S6B_1_ and S7B_1_). Similarly, co‐treatment of RPMI 8226 cells with BTZ, Rub1024, PR671A PIs and STAT3/6 INHs, or EPOX and Rub999 along with the STAT6 INH for 24 hours induced mild synergistic effects (Figures S6B_2_ and S7B_2_). The mild increase obtained in cell death in some combinations indicates a likely impact on different pathways. We then asked whether co‐treatment of MM cells with low doses (IC_10_) of the PIs and MEK 1/2 (CI‐1040) or JNK 1/2/3 (JNK‐IN‐8) inhibitors exert synergistic effects. We found that both JJN3 and RPMI 8226 cells are resistant to these inhibitors (Figure S8A); also, co‐treatment of MM cells with PIs and CI‐1040 or JNK‐IN‐8 inhibitors for 24 hours slightly increased cell death for both cell lines only in the case of co‐exposure with BTZ (Figure S8B,C).

### 3.5 Inhibition of proteasome at non‐lethal doses in MM cells results in the secretion of pro‐tumorigenic and/or immunosuppressive cytokines/chemokines

**Figure 3:**
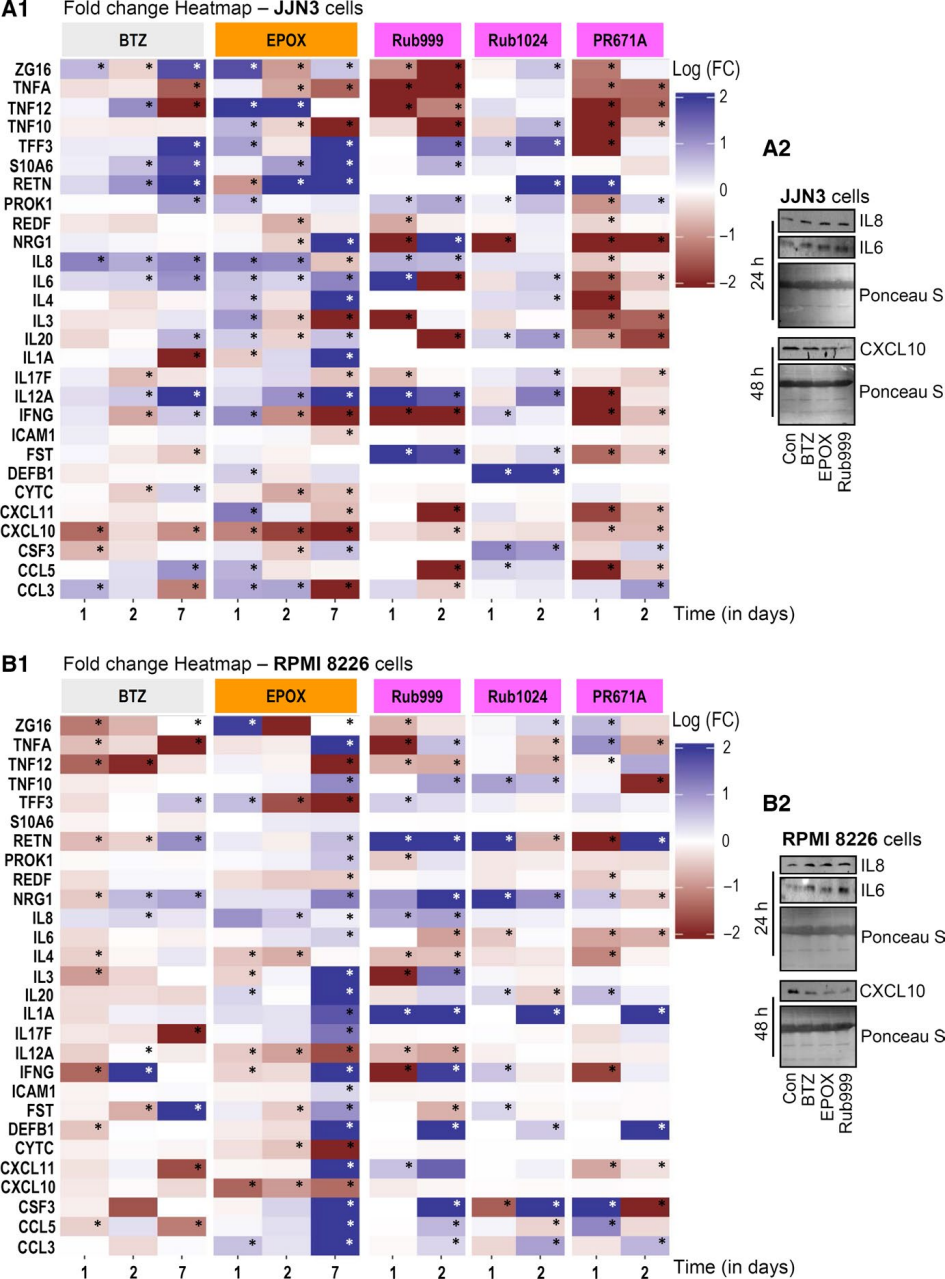
Incubation of MM cell lines with non‐lethal doses of PIs promotes (in an inhibitor‐ and cell type‐specific manner) the secretion of pro‐tumorigenic immunosuppressive molecules. A_1_, Heatmap indicating logarithmic FC values (vs control samples) of the assayed cytokine/chemokine secretion levels after treatment of JJN3 cells with either BTZ and EPOX for 24, 48 and 168 h or with Rub999, PR671A and Rub1024 for 24 and 48 h. A_2_, Representative blots of JJN3 cell culture supernatant probed with antibodies against IL6, IL8 and CXCL10 after treatment with the shown PIs for 24 or 48 h. B_1_, Heatmap indicating logarithmic FC values (vs control samples) of the assayed cytokine/chemokine secretion levels in RPMI 8226 cells treated with either BTZ and EPOX for 24, 48 and 168 h or with Rub999, PR671A and Rub1024 for 24 and 48 h. B_2_, Representative blots showing the IL6, IL8 and CXCL10 levels in RPMI 8226 cell culture supernatant after exposure to the shown PIs for 24 or 48 h. Significance (*) of the results in (A_1_, B_1_) was set as a combination of median fluorescence intensity (MFI) value above 600 and fold change (FC) value above 0.3 vs control samples. Ponceau S staining of nitrocellulose membranes in (A_2_, B_2_) was used as reference for total protein input

For these analyses by the LUMINEX platform, we used a panel of 28 cytokines/chemokines (Table S2). As for phosphoproteins, treatment of MM cells with non‐lethal doses of the PIs caused significant alterations in the secretion profile of the assayed cytokines/chemokines (Figure 3); data were also tested for their significance (asterisks in Figure 3A_1_,B_1_), plotted on bar graphs (Figure S9) and used for HCL analyses (Figure S10). In JJN3 cells, both BTZ and EPOX caused (in most cases) a gradual increase in cytokine/chemokine secretion up to day 7; yet, both PIs suppressed TNFA and CXCL10 secretion. Also, BTZ strongly decreased the secretion of TNF12 and IL1A; and EPOX of TNF10, IL3 and CCL3 at day 7. Rub999 suppressed the secretion of ZG16, TNFA, TNF12, TNF10, IL6, IL20, IFNG, CXCL11, CXCL10 and CCL5; Rub1024 caused a mild up‐regulation in the secretion of most analytes assayed, while PR671A strongly suppressed the secretion of most cytokines/chemokines studied (Figure 3A_1_; Figures S9A and S10A). At the RPMI 8226 cell line, the PIs' EPOX, Rub999, Rub1024 and PR671A showed a rather similar profile as they tended to induce the up‐regulation of cytokine/chemokine secretion, except TNF12, TFF3, IL12A, CYTC and CXCL10 for EPOX. Notably, cell exposure to BTZ caused milder (as compared to EPOX) alterations in cytokine/chemokine secretion, which in several cases (eg TNFA, TNF12, IL17F, CXCL11 and CCL5) were strongly suppressed (Figure 3B_1_; Figures S9B and S10B). Our findings for IL6, IL8 and CXCL10 were to a significant extent verified by immunoblotting analyses in JJN3 and RPMI 8226 cell lysates following treatment with the studied PIs (Figure 3A_2_,B_2_).

## 4 DISCUSSION

The use of therapeutic PIs represents a significant advance in the treatment of haematological malignancies, such as MCL and especially MM. In fact, achieving complete remission, prolonging overall survival and reaching the status of undetectable minimal residual disease are a clear triumph of most recent therapeutic interventions. Yet, the increased probability of disease relapse hinders these efforts, while the identity of the mechanisms involved remains largely elusive. Herein, we have comparatively analysed the short‐ and long‐term effects of non‐lethal doses of PIs in MM cell lines. We observed that EPOX, a CFZ‐like inhibitor, has a higher (vs BTZ) IC_50_ in the MM cell lines under study. Also, we found that selective inhibition of the C‐L or T‐L peptidases by the Rub1024 and PR671A PIs, respectively, did not exert any cytotoxicity even at high concentrations in contrast to the selective inhibition of CT‐L activity by Rub999. These findings further support the notion that the CT‐L activity is the rate limiting for protein breakdown^27^, ^28^ and, therefore, its selective inhibition triggers apoptosis. Nonetheless, as is evident by the enhanced (vs Rub999) BTZ and CFZ cytotoxicity in MM cells, the inhibition of CT‐L alone is rarely sufficient to efficiently block protein degradation. Thus, co‐inhibition of either the C‐L or the T‐L sites is required to effectively inhibit protein breakdown and to increase sensitivity of MM cells and/or other types of cancer cells (eg solid tumours) to PIs.^16^, ^19^, ^20^ In support, head‐to‐head comparison of clinically available PIs showed that in the clinically relevant setting only the co‐inhibition of C‐L or T‐L with CT‐L activity achieves meaningful functional proteasome inhibition and cytotoxicity; in this setting, the selective CT‐L/T‐L inhibition of both constitutive and immunoproteasome is the most cytotoxic.^21^

In terms of the affected cell signalling pathways, non‐lethal proteasome inhibition induced PI‐, time‐ and cell type‐specific readouts, likely due to differences in the genetic backgrounds of the cell lines under study^29^; this heterogeneity at both the genome and transcriptome levels among tumours from different patients is a MM hallmark seen also at the clinical setting.^30^ All three highly selective PIs were found to produce less intense (as compared to BTZ and EPOX) alterations in JJN3 cells or even to down‐regulate the pathways under study in RPMI 8226 cells; notably, EPOX produced a unique signature in RPMI 8226 cells, as it induced the phosphorylation of all proteins studied, presenting thus a strong pro‐tumorigenic/immunosuppressive readout. Given these findings, it is evident that any (even minor) deviation from the physiological levels of each one of the three proteasomal peptidases activity is sensed by cells and, via largely unknown mechanisms, impacts on cell signalling and immune response pathways.

BTZ and EPOX induced higher phosphorylation levels of oncogenic molecules in JJN3 cells, for example JUN, PTN11, TF65 (NF‐κB) and WNK1; it also suppressed P53 phosphorylation suggesting a switch towards increased proliferation and survival. For BTZ and EPOX, NF‐κB activation coincides with reduced phosphorylation of its inhibitor IKBA indicating that the pathway is indeed activated. A similar, yet weaker, NF‐κB response was also evident for the three selective PIs studied. NF‐κB has been linked to bone marrow microenvironment alterations, cell growth and drug resistance in tumour cells^31^, ^32^, ^33^; notably, NF‐κB was also activated in RPMI 8226 cells after EPOX treatment. From all the analytes studied, the most consistent response across cell lines, PIs and duration of treatment was the notable activation of STAT3 and STAT6. STAT3 is closely associated with inflammation, tumorigenesis and MM cell survival^34^ and it is induced by IL6^35^ which, as we found herein, is over‐secreted following non‐lethal proteasome inhibition in MM cells. STAT3 has been associated with poor survival of MM patients^36^ and resistance to lenalidomide,^37^ while its inhibition suppressed MM cell growth^38^ suggesting that it represents a promising therapeutic target in MM.^39^ Consistently, it was found that MM exosomes establish a favourable bone marrow microenvironment which enhanced angiogenesis and immunosuppression through activation of the STAT3 pathway,^40^ as well as that STAT3 establishes an immunosuppressive microenvironment during the early stages of breast carcinogenesis to promote tumour growth and metastasis.^41^ Similarly, mounting evidence for STAT6, in both patients and mouse models, supports a model where STAT6 is not a mere bystander, but rather, plays an active role in promoting a transformed phenotype in various types of cancer,^42^ including also the establishment of an immunosuppressive tumour microenvironment.^43^ Furthermore, activated STAT3 and STAT6 cooperate in tumour‐associated macrophages to promote a secretory phenotype that enhances tumour progression.^44^

These findings largely coincide with our cytokine/chemokine profiling after non‐lethal proteasome inhibition in MM cells. Again, the readout was PI‐ and cell type‐specific, as BTZ and EPOX induced the secretion of almost all mediators studied in JJN3 cells; these effects were either milder or inverted for the selective PIs studied, indicating that the combined suppression of more than one proteasome peptidases induces unique responses as compared to peptidase‐selective inhibition. Similarly, to alterations in cell signalling, these patterns were different in RPMI 8226 cells, where responses were in most cases (except treatment with EPOX at day 7) indicative of reduced cytokine/chemokine secretion. Again, the observed cell line‐specific responses can be attributed to the different genetic backgrounds of the MM cell lines studied, to different patterns of proteasome peptidase inhibition or reversibility of PIs' binding to proteasome (see above), as well as to distinct off‐target effects of the PIs.

Among the found responses, the secretion of TNFA and CXCL10 was suppressed, whereas that of IL6 and IL8 was induced in a PI‐, time‐ and cell type‐independent manner. It has been reported that TNF‐related apoptosis‐inducing ligand (TRAIL)‐armed exosomes deliver proapoptotic signals to the tumour site,^45^ while elotuzumab enhances natural killer cell activation and myeloma cell killing through IL2‐ and TNFA‐mediated pathways.^46^ Also, several lines of evidence support the role of the potent T cell chemoattractant CXCL10 in restraining cancer development.^47^ Specifically, in addition to its role in inducing TH1‐type effector cells, CXCL10 was recently associated with the recruitment of CXCR3^+^/CD8^+^ T cells to the tumour site and also with the induction of granzyme‐B production by these cells, thereby potentiating their antitumour activities.^48^ It was thus suggested that CXCL10 stabilization (eg a CXCL10‐Ig fusion protein) can be used to stimulate anticancer immunity.^47^ Also, heparinase enhanced myeloma progression via CXCL10 down‐regulation^49^ and its plasma levels correlated with survival and chemotherapeutic efficacy in advanced pancreatic ductal adenocarcinoma.^50^

On the other hand, IL6, a STAT3 activator, has a pleiotropic effect on inflammation, immune response and haematopoiesis and is involved in the survival and proliferation of MM cells.^51^ Notably, IL6 is implicated in chemotherapy resistance by regulating the activity of anti‐apoptotic heat shock proteins and siltuximab, a chimeric mAb against IL6, is being tested in various clinical studies along with PI treatment.^52^ IL6 levels predict event‐free survival in paediatric AML suggesting a mechanism of chemotherapy resistance^53^ and IL32α promotes the proliferation of MM cells by inducing production of IL6 in bone marrow stromal cells.^54^ Consistently, glioblastoma‐derived IL6 induces immunosuppressive peripheral myeloid cell PD‐L1 and promotes tumour growth^55^; also, in the tumour microenvironment of upper‐gastrointestinal cancers, IL6 mediates the cross‐talk between tumour cells and pro‐tumorigenic activated fibroblasts.^56^ Similarly, IL8 is implicated in cancer cell growth, survival, angiogenesis and metastasis in several tumours.^57^ In support, bone marrow plasma and stromal cells from patients with MM were found to secret higher amounts of IL8 than healthy donors; additionally, IL8 up‐regulation is involved in MM bone disease and bone marrow angiogenesis.^58^ Notably, proteasome inhibition increased recruitment of IκB kinase β, S536P‐p65, and transcription factor EGR1 to the IL‐8 promoter, resulting in increased IL8 production in ovarian cancer cells.^59^

Taken together, our findings indicate that those MM cells that survive treatment with therapeutic PIs likely shape a pro‐tumorigenic immunosuppressive cellular and secretory bone marrow microenvironment that enables malignancy to relapse (Table S3). Also, they reveal new opportunities for combinatorial therapeutic interventions in MM and/or other haematological malignancies by employing inhibitors of STATs and/or secretory cytokines/chemokines. Consistently with the notion that the dynamic milieu generated by the cytokines/chemokines as a whole may dictate treatment response and disease outcome, recent studies have revealed that combinatorial therapies with PIs plus anticytokine/chemokine (eg anti‐IL6) treatment could have beneficial effect on MM therapy and on MM‐related bone disease.^60^

## CONFLICT OF INTEREST

The authors declare no conflict of interest.

## AUTHOR CONTRIBUTIONS

AS, ADS and DDG conducted experimental work; TS and LGA performed the phosphoproteomic and cytokine profiling analyses; BIF and HSO synthesized PIs; ET, EK, OT and MAD generated or contributed reagents, materials and analysis tools; IPT designed, supervised the study and wrote the manuscript. All authors interpreted data and commented on the manuscript.

## Data Availability

The data sets generated and/or analysed during the current study are available from the corresponding author on reasonable request.
