# Supplementary material for: Non‐lethal proteasome inhibition activates pro‐tumorigenic pathways in multiple myeloma cells
Source: J Cell Mol Med. 2019 Sep 30;23(12):8010–8. doi: 10.1111/jcmm.14653 (PMC6850931; doi:10.1111/jcmm.14653)
Supplement: Supplementary file 1 [file JCMM-23-8010-s001.pdf]

Non-lethal proteasome inhibition activates pro-tumorigenic pathways in multiple myeloma cells

Aikaterini Skorda*, Aimilia D. Sklirou*, Theodore Sakellaropoulos, Despoina D. Gianniou, Efstathios Kastritis, Evangelos Terpos, Ourania E. Tsitsilonis, Bogdan I. Florea, Herman S. Overkleeft, Meletios A. Dimopoulos, Leonidas G. Alexopoulos and Ioannis P. Trougakos

Supporting Information

Supporting Figures

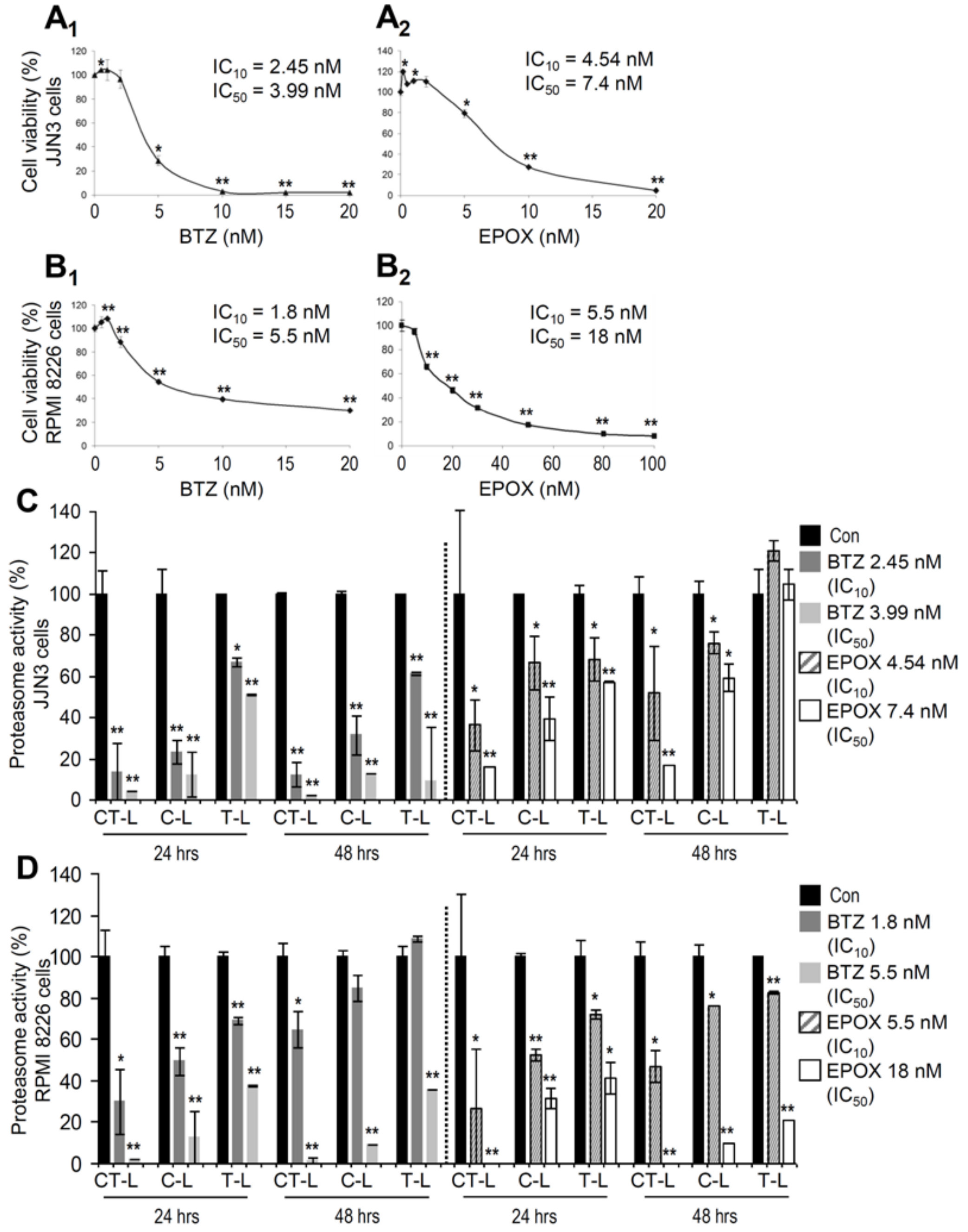

Figure S1. BTZ and EPOX induce significant cell death and proteasome peptidases activity inhibition at MM cell lines. (A, B) Relative (%) viability of JJN3 (A) and RPMI 8226 (B) MM cell lines exposed to BTZ and EPOX at the indicated concentrations for 24 h. (C, D) Relative proteasome peptidases activities (CT-L, C-L and T-L) in JJN3 (C) and RPMI 8226 (D) cells treated for 24 or 48 h with BTZ and EPOX at IC_{10} and IC_{50} concentrations (as indicated in the graph legends). Bars: ± SD, *: $p<0.05$, **: $p<0.01$ *vs*. controls set to 100%.

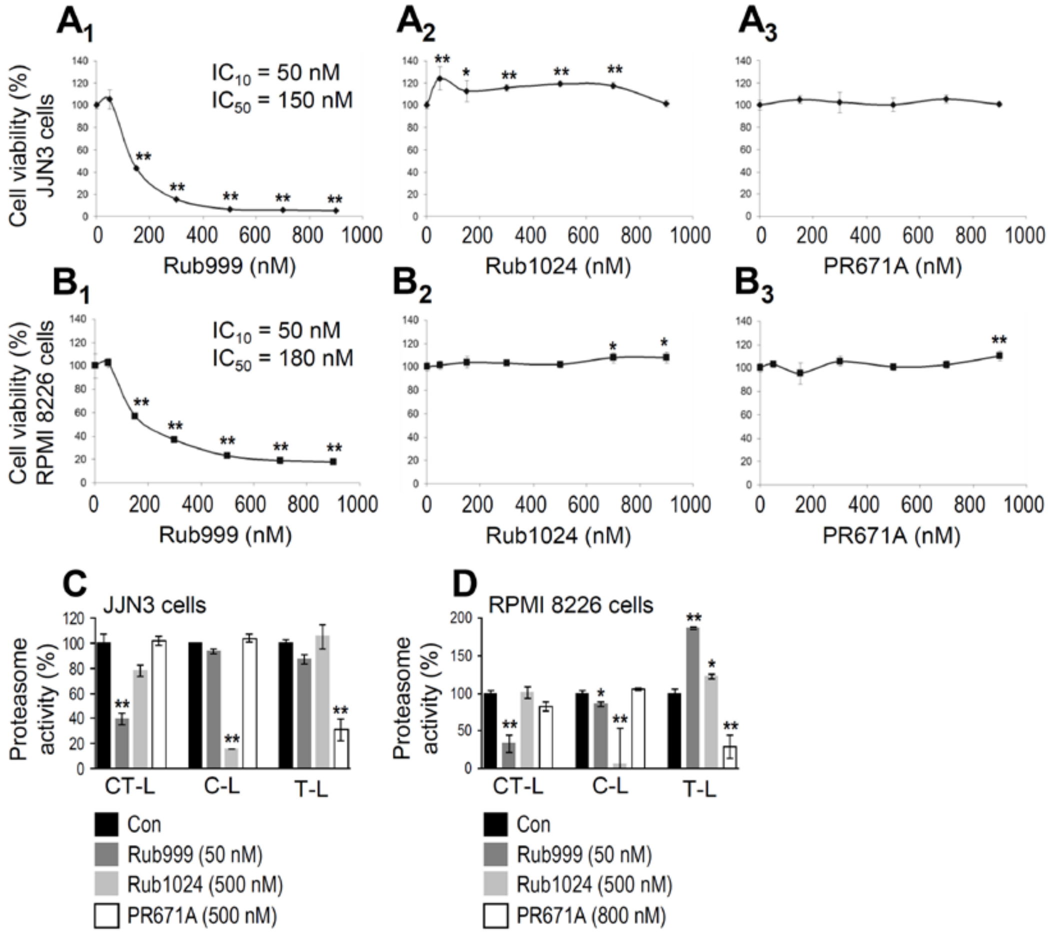

Figure S2. Effects of highly selective for specific proteasome peptidases PIs on MM cell lines viability and proteasome activities. (A, B) Relative (%) cell survival of JJN3 (A) and RPMI 8226 (B) MM cells after treatment with Rub999, Rub1024 and PR671A PIs at the indicated concentrations for 24 h. (C, D) Relative activity of the CT-L, C-L and T-L proteasome peptidases in JJN3 (C) and RPMI 8226 (D) cell lines after incubation with the PIs Rub999, Rub1024 and PR671A at the indicated concentrations for 24 h. Bars: ± SD, *: $p<0.05$, **: $p<0.01$ *vs*. controls set to 100%.

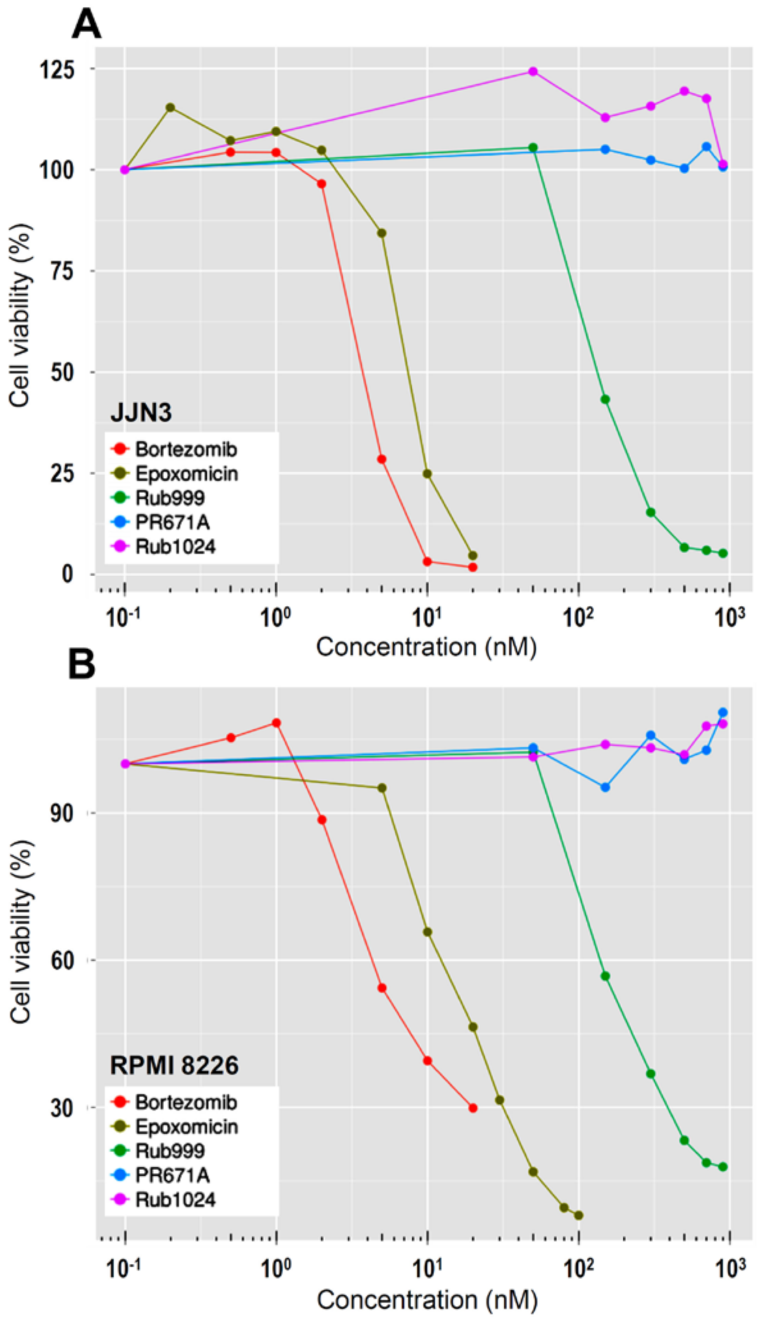

Figure S3. Comparative analyses of cell viability after exposing MM cell lines to different concentrations of the PIs under study. Relative (%) survival of JJN3 (A) and RPMI 8226 (B) cells after incubation with the PIs BTZ, EPOX, Rub999, PR671A or Rub1024 at the indicated concentrations for 24 h.

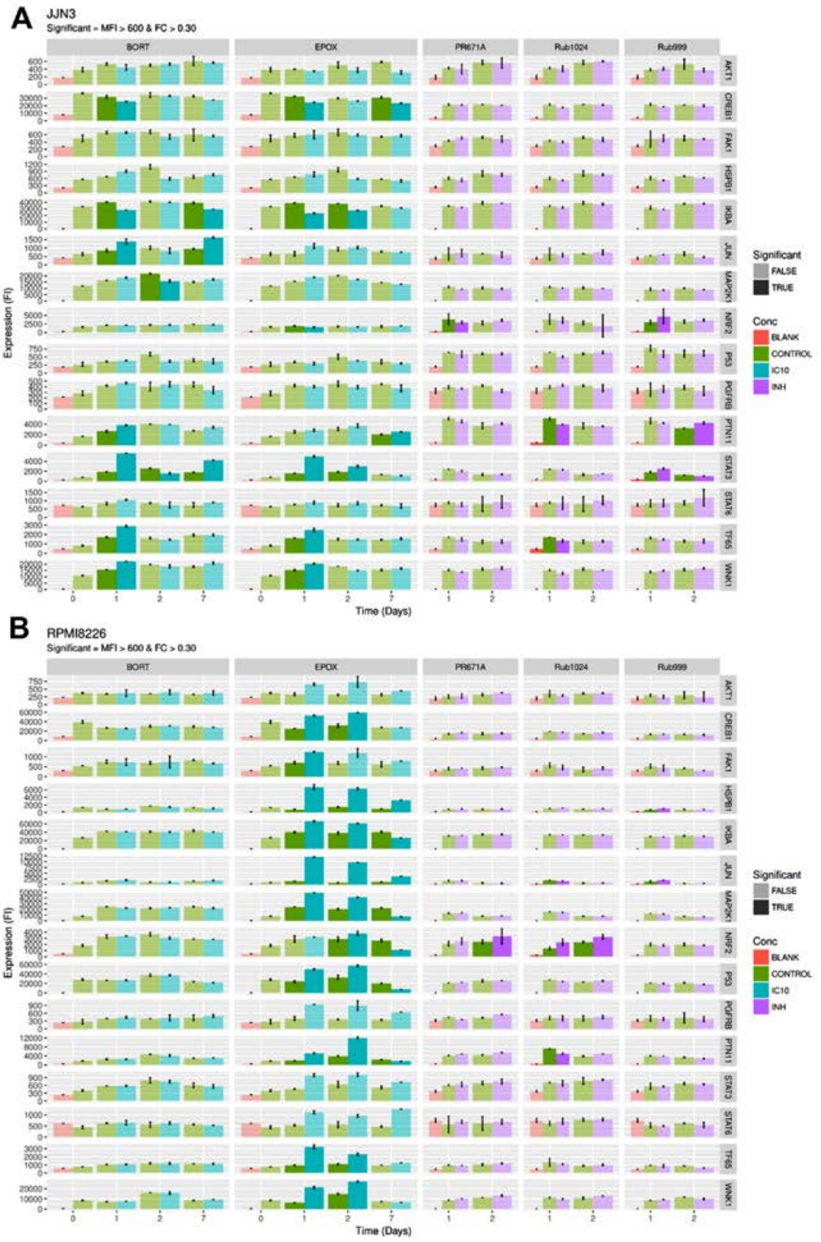

Figure S4. Partial proteasome inhibition induces cell signalling pathways alterations in MM cell lines. Relative phosphorylation levels of proteins participating in major signalling pathways in MM cell lines JJN3 (A) and RPMI 8226 (B) exposed to either BTZ and EPOX for 24, 48 and 168 h or to Rub999, PR671A and Rub1024 for 24 and 48 h; BORT refers to BTZ. Each small subplot represents the changes observed in the phosphorylation status of a specific protein compared to the control samples through different time points. Significance of the results is set as a combination of Median Fluorescence Intensity (MFI) value above 600 and Fold Change (FC) value above 0.3 when compared to the control samples.

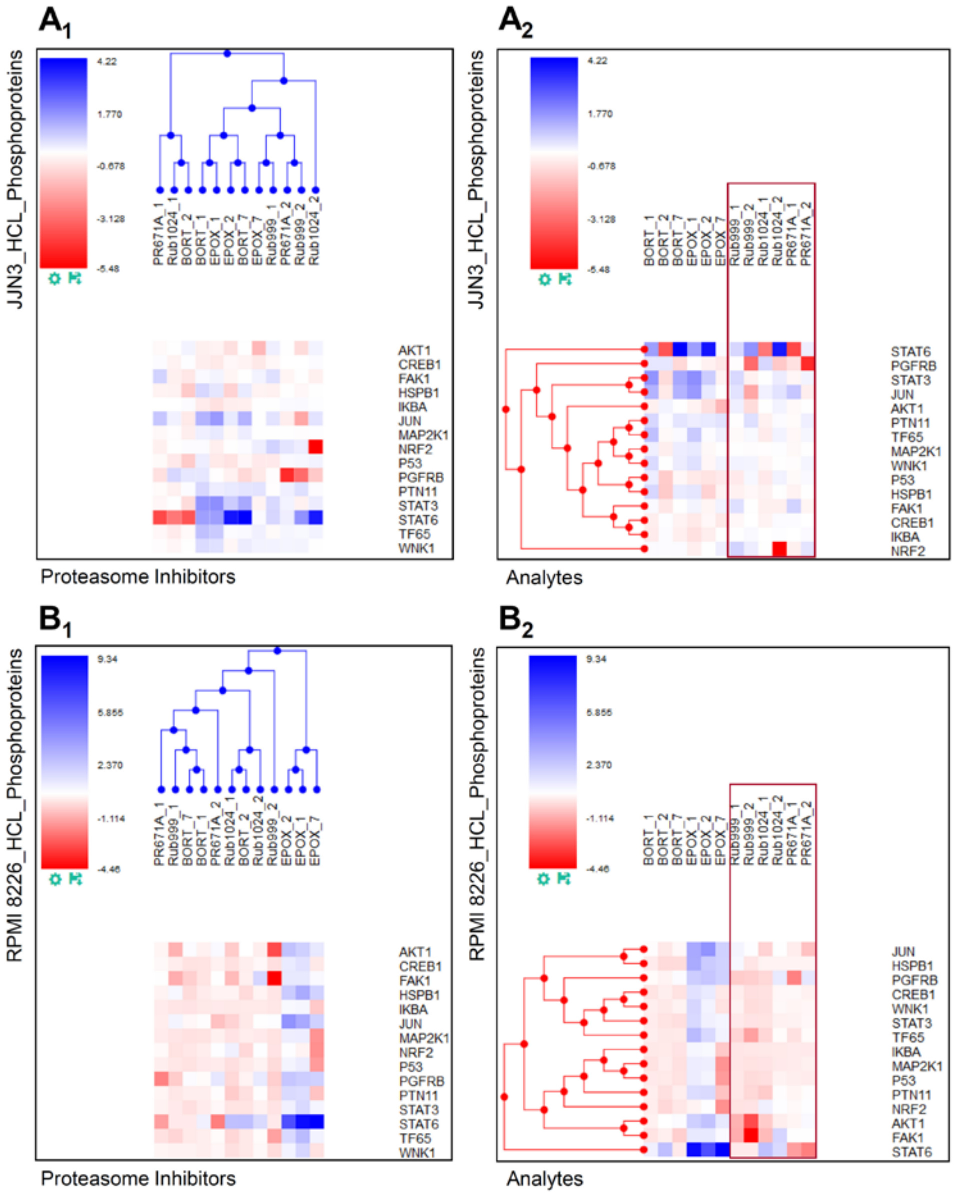

Figure S5. Hierarchical Clustering (HCL) of phosphoproteomic profiling in MM cells. (A) HCL for PIs (A_1) or analytes (phosphoproteins) (A_2) in JJN3 cells. (B) HCL for PIs (B_1) or analytes (phosphoproteins) (B_2) in RPMI 8226 cells. HCL was done by using the MEV tool (http://mev.tm4.org/#/welcome); BORT refers to BTZ. Numbers after each PI indicate days of treatment. Distance Metrics: Euclidean; Linkage criteria algorithm: Complete.

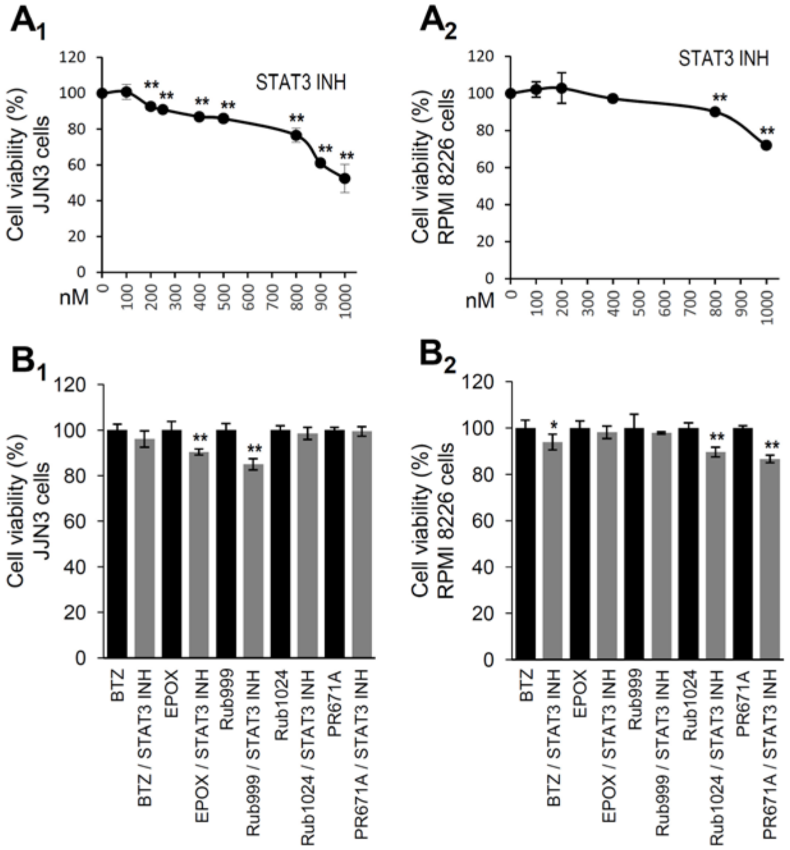

Figure S6. Proteasome inhibition along with STAT3 blockage mildly enhanced (in some combinations) cell death in MM cells lines. (A_1, A_2) Relative (%) viability of JJN3 (A_1) and RPM1 8226 (A_2) cell lines incubated with the STAT3 inhibitor (STAT3 INH) Stattic at the shown concentrations for 24 h. (B_1, B_2) Relative (%) survival of JJN3 (B_1) and RPMI 8226 (B_2) cells exposed to PIs (at IC_{10} concentration) in the presence or absence of the STAT3 INH for 24 h. The (%) cell viability following combined treatment of PIs plus the STAT3 inhibitor was *vs*. the values obtained after the sole treatment of cells with each one of the shown PIs. BTZ (JJN3 cells, 2.45 nM; RPMI 8226, 1.8 nM), EPOX (JJN3 cells, 4.54 nM; RPMI 8226, 5.5 nM), Rub999 (JJN3 and RPMI 8226 cells, 50 nM), Rub1024 (JJN3 and RPMI 8226 cells, 500 nM), PR671A (JJN3 cells, 500 nM; RPMI 8226 cells, 800 nM), Stattic (JJN3 cells, 250 nM; RPMI 8226, 1.1 μM). Bars: ± SD, *: $p<0.05$, **: $p<0.01$ *vs*. controls set to 100%.

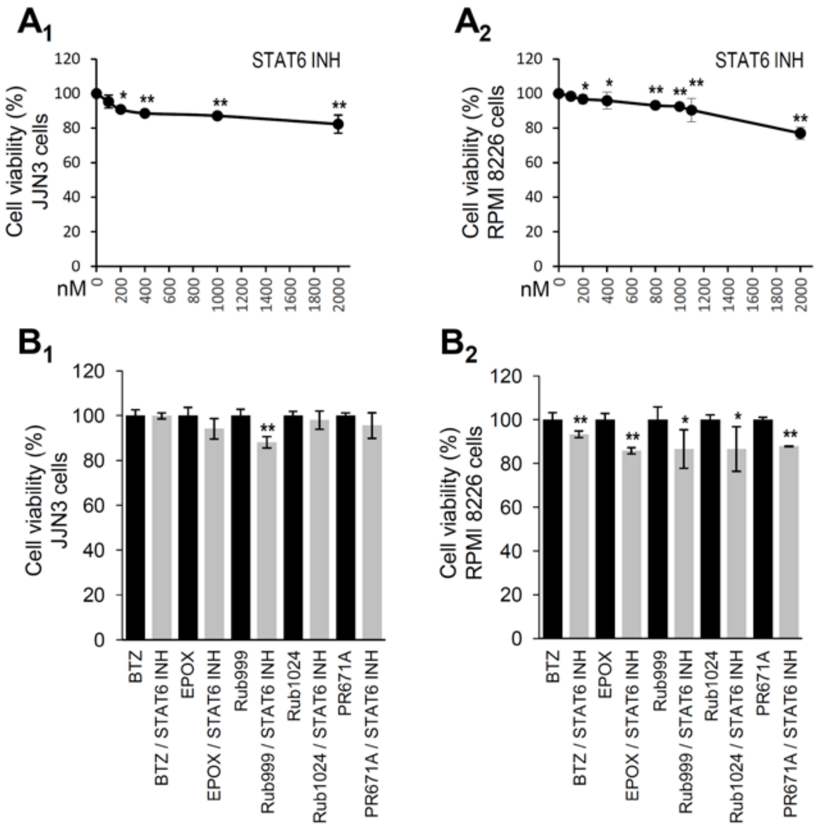

Figure S7. Combined proteasome and STAT6 inhibition only mildly increased (in some combinations) toxicity of PIs in MM cells. (A_1, A_2) Relative (%) viability of JJN3 (A_1) and RPM1 8226 (A_2) cell lines incubated with the STAT6 inhibitor (STAT6 INH), AS1517499 at the shown concentrations for 24 h. (B_1, B_2) Relative (%) survival of JJN3 (B_1) and RPMI 8226 (B_2) cells exposed to PIs (at IC_{10} concentration) in the presence or absence of the STAT6 INH for 24 h. The (%) cell viability following combined treatment of PIs plus the STAT6 inhibitor was *vs*. the values obtained after the sole treatment of cells with each one of the shown PIs. BTZ (JJN3 cells, 2.45 nM; RPMI 8226, 1.8 nM), EPOX (JJN3 cells, 4.54 nM; RPMI 8226, 5.5 nM), Rub999 (JJN3 and RPMI 8226 cells, 50 nM), Rub1024 (JJN3 and RPMI 8226 cells, 500 nM), PR671A (JJN3 cells, 500 nM; RPMI 8226 cells, 800 nM), AS1517499 (JJN3 cells, 200 nM; RPMI 8226, 800 nM). Bars: ± SD, *: $p<0.05$, **: $p<0.01$ *vs*. controls set to 100%.

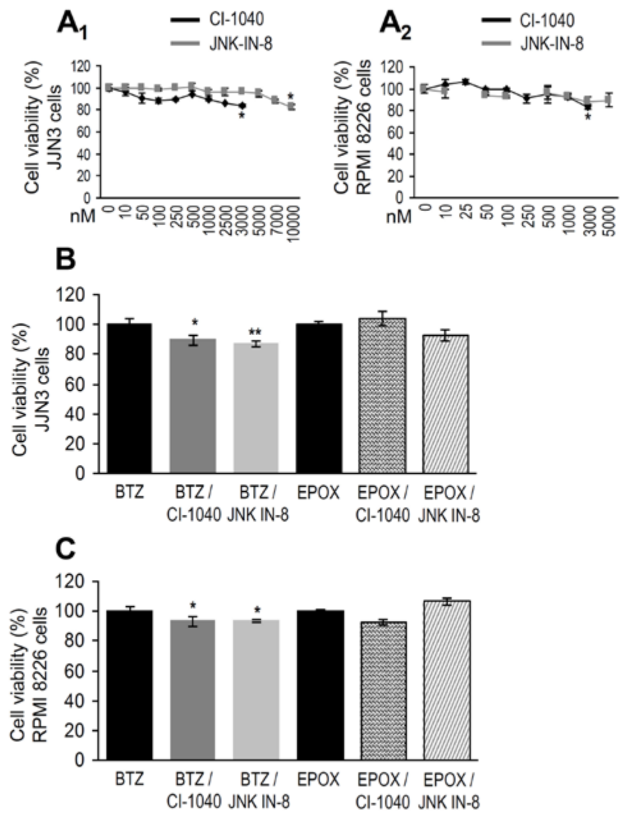

Figure S8. Combined treatment of MM cells with PIs and inhibitors of MAPK pathway did not significantly enhance toxicity of PIs. (A_1, A_2) Relative (%) viability (MTT assay) of JJN3 (A_1) and RPMI 8226 (A_2) cell lines after exposure to increasing concentrations of MEK 1/2 (CI-1040) and JNK 1/2/3 (JNK-IN-8) inhibitors for 24 h. (B, C) Relative (%) survival of JJN3 (B) and RPMI 8226 (C) cells exposed to BTZ or EPOX (at IC_{10} concentration) in the presence or absence of CI-1040 or JNK-IN-8 inhibitors for 24 h. BTZ (JJN3 cells, 2.45 nM; RPMI 8226, 1.8 nM), EPOX (JJN3 cells, 4.54 nM; RPMI 8226, 5.5 nM), CI-1040 (JJN3 cells, 50 nM; RPMI 8226, 1.75 μM), JNK-IN-8 (JJN3 cells, 6 μM; RPMI 8226, 2 μM). Bars: ± SD, *: $p<0.05$, **: $p<0.01$ *vs*. controls set to 100%.

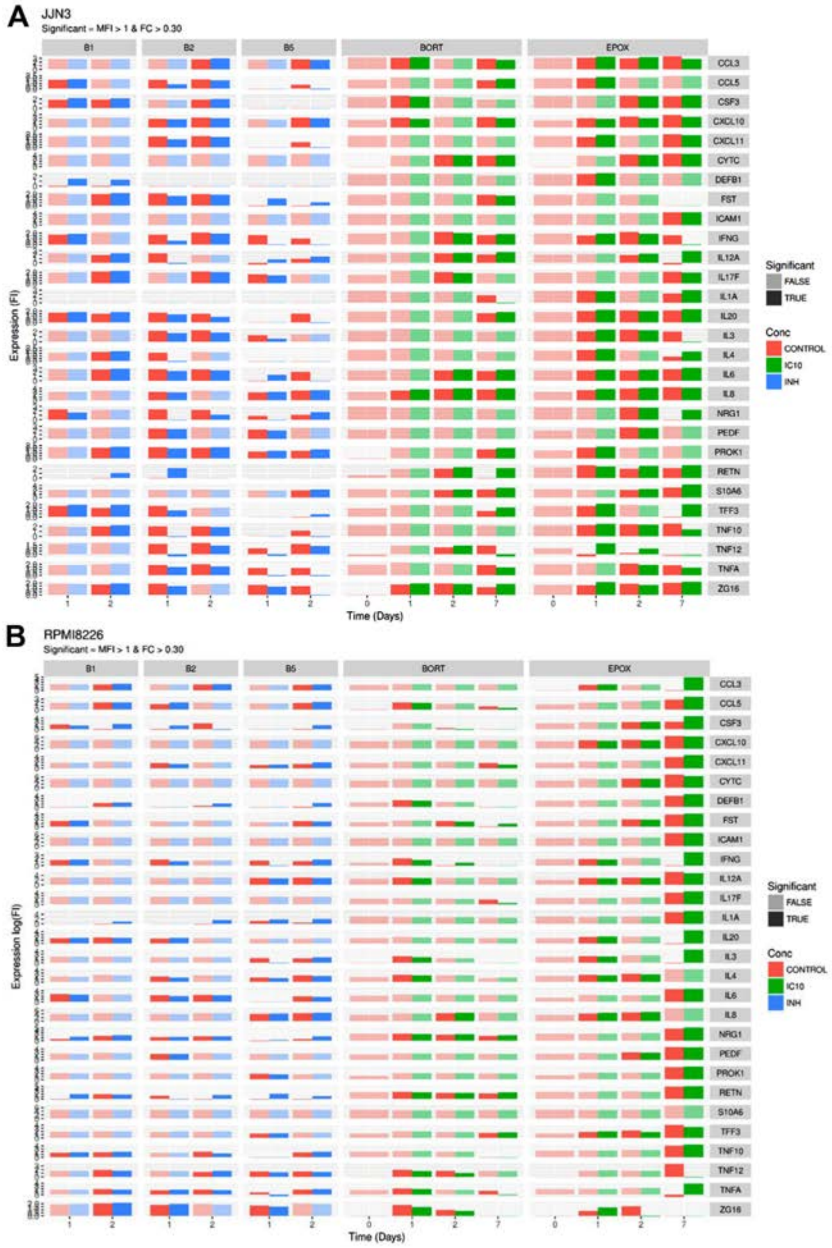

Figure S9. Secretory profile of cytokines/chemokines upon treatment of MM cell lines with non-lethal doses of PIs. Relative levels of cytokines/chemokines secretion in MM cell lines JJN3 (A) and RPMI 8226 (B) exposed to either BTZ and EPOX for 24, 48 and 168 h or to Rub999, PR671A and Rub1024 for 24 and 48 h. Each small subplot represents the changes observed in the secretion pattern of a specific protein compared to the control samples through different time points. Significance of the results is set as a combination of Median Fluorescence Intensity (MFI) value above 600 and Fold Change (FC) value above 0.3 when compared to the control samples. BORT refers to BTZ, whereas B1, B2 and B5 refer to Rub1024, PR671A and Rub999, respectively.

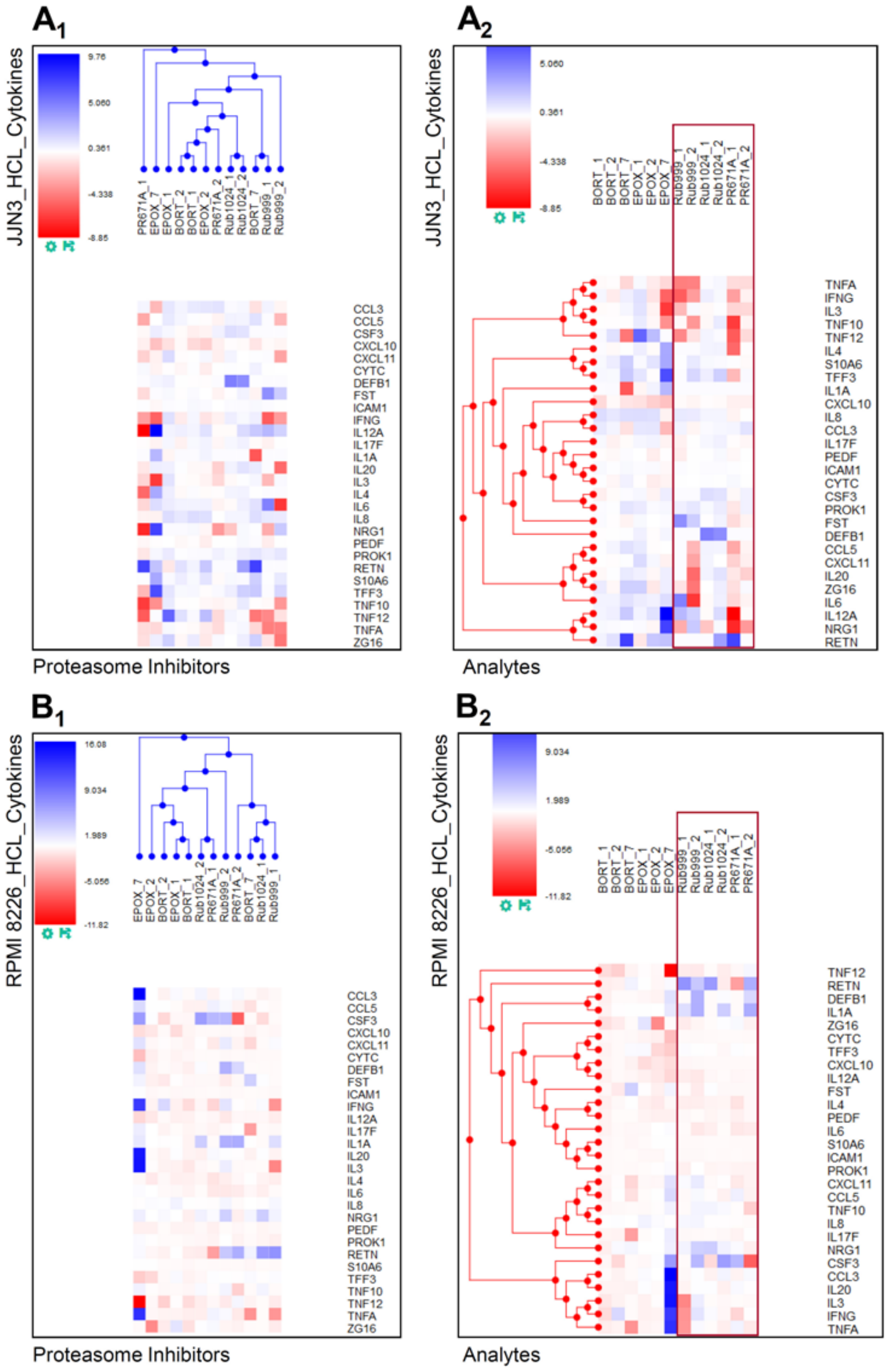

Figure S10. Hierarchical Clustering (HCL) of cytokines/chemokines profiling in MM cells. (A) HCL for PIs (A_1) or analytes (cytokines/chemokines) (A_2) in JJN3 cells. (B) HCL for PIs (B_1) or analytes (cytokines/chemokines) (B_2) in RPMI 8226 cells. HCL was done by using the MEV tool (http://mev.tm4.org/#/welcome); BORT refers to BTZ. Numbers after each PI indicate days of treatment. Distance Metrics: Euclidean; Linkage criteria algorithm: Complete.

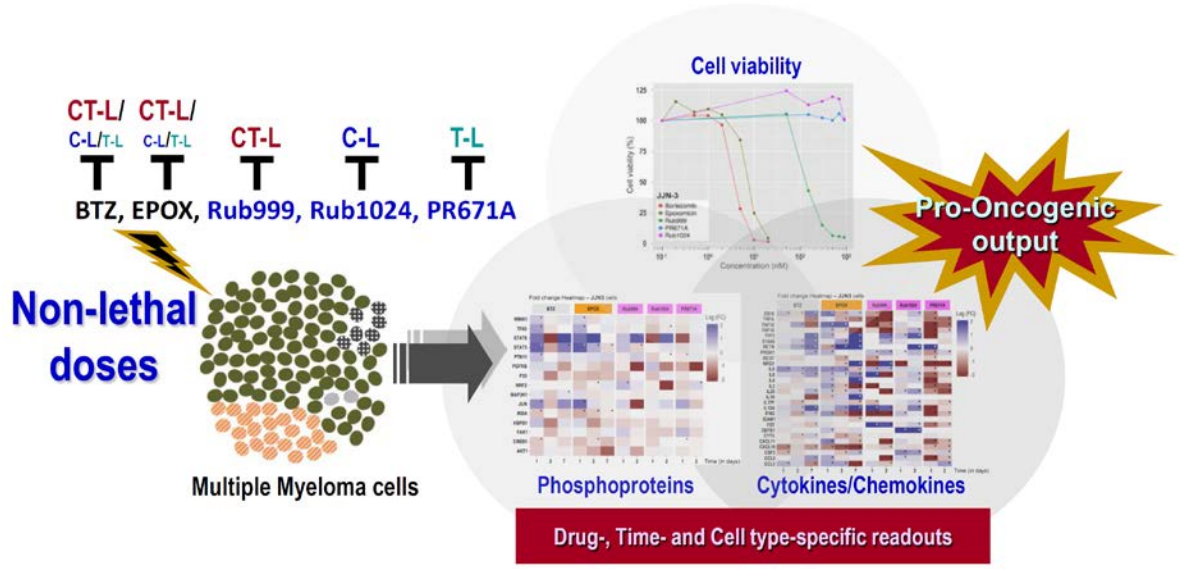

Graphical Abstract

PROTEIN	GENE NAME	FUNCTION	PHOSPHO-RESIDUE	EFFECT
**WNK1**	*WNK1*	Regulation of electrolyte homeostasis, cell signaling, survival and proliferation	T60	ACTIVATION
**TF65**	*RELA*	Involvement in inflammation, immunity, differentiation, cell growth, tumorigenesis and apoptosis	S536	ACTIVATION
**STAT6**	*STAT6*	Signal transduction and transcripton activation, involvement in IL-3 and IL-4 mediated signaling	Y641	ACTIVATION
**STAT3**	*STAT3*	Signal transduction and transcripton activation, mediation of cellular responces to interleukins and other growth factors	Y705	ACTIVATION
**PTN11**	*PTPN11*	Participation in signal transduction from cell surface to the nucleus, positive regulation of the MAPK pathway	Y542	ACTIVATION
**PGFRB**	*PDGFRB*	Activates PIK3R1 leading to AKT1 activation; phosporylation of SHC1 activates HRAS, RAF1 and MAP kinases	Y751	ACTIVATION
**P53**	*P53*	Tumor suppression, induction of growth arrest or apoptosis, cell cycle regulation	S46	ACTIVATION
**NRF2**	*NFE2L2*	Transcription activation by binding to ARE elements in the promoter regions of target genes, up-regulation of genes in response to oxidative stress	S40	ACTIVATION
**MAP2K1**	*MAP2K1*	Mediation of cell growth, adhesion, survival and differentiation, part of the MAPK/ ERK cascade	S217/S221	ACTIVATION
**JUN**	*JUN*	Involvement in increased steroidogenic gene expression upon cAMP signaling pathway stimulation	S63	ACTIVATION
**IKBA**	*NFKBIA*	Promotion of ubiquitination and degradation, enabling the dimeric RELA to translocate to the nucleus and activate transcription	S32/S36	SUPPRESSION
**HSPB1**	*HSPB1*	Molecular chaperon, maintaining denatured proteins in folding competent state, role in stress resistance and actin organization	S78/S82	ACTIVATION
**FAK1**	*PTK2*	Promotes activation of phosphatidylinositol 3-kinase, the AKT1 signaling cascade, MAPK1/ERK2, MAPK3/ERK1 and the MAP kinase signaling cascade	Y397	ACTIVATION
**CREB1**	*CREB1*	Stimulation of transcription upon binding to the DNA cAMP response element (CRE), synchronization of circadian rhythmicity and the differentiation of adipose cells	S133	ACTIVATION
**AKT1**	*AKT1*	Regulation of many processes including metabolism, proliferation, cell survival, growth and angiogenesis	S473	ACTIVATION

Supporting Table S1. Phosphoproteins assayed (including a short description of their function; source: www.uniprot.org), phosphorylated residues and activation status.

CYTOKINE / CHEMOKINE	GENE NAME	FUNCTION
**ZG16**	*ZG16*	Potentional role in protein trafficking
**TNFA**	*TNF*	Induction of cell death of certain tumor cell lines, potent pyrogen, inducton of cachexia, stimulation of cell proliferation and induction of cell differentiation
**TNF12**	*TNFSF12*	Weak induction of apoptosis in some cell lines, mediation of NFKB activation, promotion of angiogenesis and proliferation of endothelial cells, induction of inflammatory cytokines, promotion of IL8 secretion
**TNF10**	*TNFSF10*	Induction of apoptosis
**TFF3**	*TFF3*	Involvement in the maintenance and repair of the intestinal mucosa, promotion of the mobility of the epithelial cells in healing processes
**S10A6**	*S100A6*	Calcium sensor and modulator contributing to celular calcium signaling, reorganization of the actin cytoskeleton, induction of cell motility
**RETN**	*RETN*	Suppression of insulin ability to stimulate glucose uptake into adipose cells, promotion of chemotaxis in myeloid cells, pro-inflammatory
**PROK1**	*PROK1*	Induction of proliferation, migration and fenestration of capillary endothelial cells derived from endocrine glands
**PEDF**	*PEDF*	Induction of extensive neuronal differentiation in retinblastoma cells, potential inhibition of angiogenesis
**NRG1**	*NRG1*	Stimulation of tyrosine phosphorylation and activaton of ERBB receptors, induction of growth and differentiation of epithelial, glial, neuronal and skeletal muscle cells, induction of the phosphorylation and activation of MAPK3/ERK1, MAPK1/ERK2 and AKT1
**IL8**	*CXCL8*	Attraction of neutrophils, basophils and T-cells, involvement in neutrophil activation, released in response to inflammatory stimulus
**IL6**	*IL6*	Potent induction of the acute phase response, involvement in B-cell, lymphocyte and monocyte differentiation, induction of myeloma and plasmacytoma growth and nerve cells differentiation
**IL4**	*IL4*	Co-stimulator of DNA-synthesis, induction of the expression of class II MHC molecules on resting B-cells
**IL3**	*IL3*	Involvement in hematopoiesis by controling the production, differentiation and function of granulocytes and macrophages
**IL20**	*IL20*	Proinflammatory and angiogenic, may act through STAT3
**IL1A**	*IL1A*	Stimulation of thymocyte proliferation, B-cell maturation and proliferation and fibroblast growth factor activity, involvement in inflammatory responses
**IL17F**	*IL17F*	Stimulation and production of IL6, IL8 and CSF2, stimulation of proliferation of PBMCs and T-cell, inhibition of angiogenesis
**IL12A**	*IL12A*	Growth factor for activated T and NK cells, enhancement of the lytic activity of killer cells, stimulation of IFN-γ production by resting PBMCs
**IFNG**	*IFNG*	Possesion of antiviral activity, potent activator of macrophages, antiproliferative effects on transformed cells, potential antiviral and antitumor effects
**ICAM1**	*ICAM1*	Intercellular adhesion molecule 1, ligand for the leukocyte adhesion protein LFA-1
**FST**	*FST*	Inhibition of the bosynthesis and secretion of FSH, activin antagonist
**DEFB1**	*DEFB1*	Possesion of bactericidal activity, positive regulation of sperm motility
**CYTC**	*CST3*	Inhibition of cysteine proteinases
**CXCL11**	*CXCL11*	Induction of calcium release in activated T-cells; potential role in skin immune responces
**CXCL10**	*CXCL10*	Chemotactic for interleukin-activated T-cells, and induction of calcium release; attachment to CXCR3
**CSF3**	*CSF3*	Involvement in hematopoiesis by controling the production, differentiation and function of granulocytes and macrophages
**CCL5**	*CCL5*	Chemoatrractant for blood monocytes, memory T-heper cells and eosinophils. Induction of the release of histamine from basophils and activation of eosinophils
**CCL3**	*CCL3*	Inflammatory and chemokinetic properties, major HIV-suppressive factor produced by CD8+ T-cells

Supporting Table S2. Cytokines/chemokines assayed (including a short description of their function; source: www.uniprot.org).

	BTZ	EPOX	Rub999
**JJN3**	↑ p-STAT6<br>↑ p-STAT3<br><br>↑ IL8<br>↑ IL6<br>↓ CXCL10	↑ p-STAT6<br>↑ p-STAT3<br><br>↑ IL8<br>↑ IL6<br>↓ CXCL10	↑ p-STAT6<br>↑ p-STAT3<br><br>↑ IL8<br>↑ IL6<br>↓ CXCL10
**RPMI 8226**	↑ p-STAT6<br>(*nc*) p-STAT3<br><br>↑ IL8<br>↓ IL6<br>↓ CXCL10	↑ p-STAT6<br>↑ p-STAT3<br><br>↑ IL8<br>↑ IL6<br>↓ CXCL10	(*nc*) p-STAT6<br>(*nc*) p-STAT3<br><br>↑ IL8<br>↓ IL6<br>↓ CXCL10

Supporting Table S3. Summary of the major trends noted regarding phosphorylation (p-STAT3, p-STAT6) or secretion (IL6, IL8, CXCL10) of the shown molecules in JJN3 and RPMI 8226 cells following treatment with non-lethal doses of BTZ, EPOX or Rub999; the indicated alterations may fluctuate during prolonged treatment. *nc*: no significant change.

Supporting Materials and Methods

Cell viability assay

The cytotoxic effects of the PIs against the MM cell lines were determined by using the MTT reagent (Sigma-Aldrich). Specifically, 8,000 cells per well were plated in flat-bottomed 96-well plates with the use of phenol red free RPMI 1640 medium (Lonza), in the presence (or not) of different concentrations of PIs, MAPK and STAT inhibitors or the MTH1 inhibitor. The final volume was adjusted to 100 μl per well. The plates were transferred in a humidified incubator for 24 h at 37°C. Subsequently, 10 μl of MTT solution (5 mg/ml in PBS) was added in each well. After 3 to 4 h of incubation, 100 μl of solubilization solution (10% SDS in 0.01N HCl) was added to dissolve the formazan crystals and the plates were incubated overnight at 37°C. Plates were then sufficiently stirred and the optical density (OD) at 570 nm wavelength was measured. The percentage of cell viability was calculated by setting OD obtained by control samples (absence of PIs) to 100%.

Measurement of proteasome peptidases activities

For proteasome activities measurement, cells were plated in 35 mm petri dishes at a concentration of 500,000 cells per ml, in the presence (or not) of the selected concentrations of PIs. After 24 h to 48 h treatment, cells were collected, lysed on ice and proteasome activities were measured as described before[18]. The values obtained by control samples (no PI addition) were set as 100% peptidase activity.

Immunoblotting analysis and antibodies used

Immunoblotting analysis was performed in cell culture supernatants and in cell lysates as described before[18]. Primary antibodies against p-STAT3 (9145) and p-STAT6 (9361), as well as against IL6 (500-P26G) were purchased from Cell Signaling Technology and PeproTech, Inc, respectively. The antibodies against IL8 (sc-376750), CXCL10 (sc-101500) and GAPDH (sc-25778) were obtained from Santa Cruz Biotechnology. The secondary HRP-conjugated IgG antibody was purchased from Jackson ImmunoResearch Laboratories, Inc.

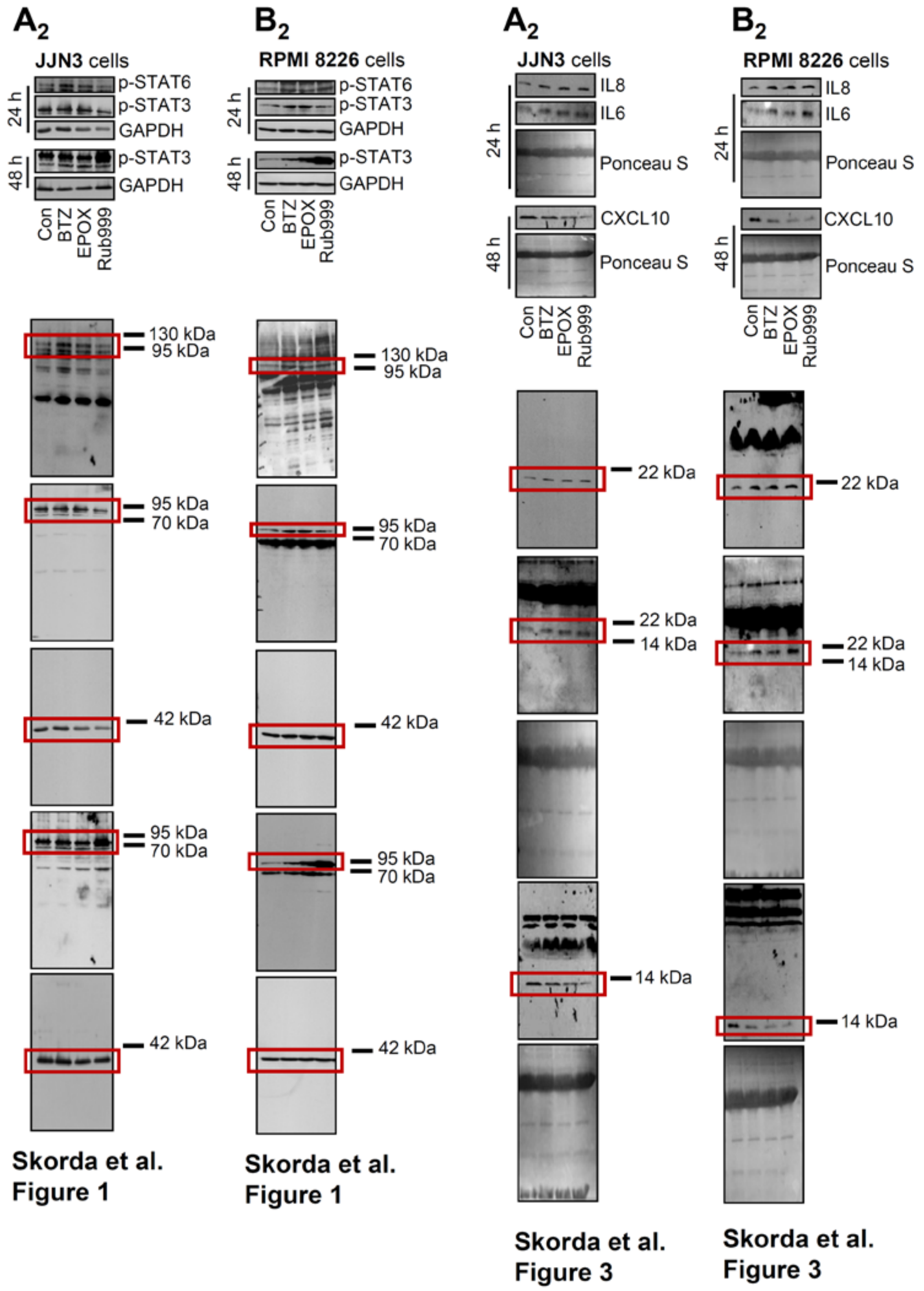

Full blots of the immunoblotting assays performed.
